# Reference Gene Selection and Validation for the Early Responses to Downy Mildew Infection in Susceptible and Resistant *Vitis vinifera* Cultivars

**DOI:** 10.1371/journal.pone.0072998

**Published:** 2013-09-04

**Authors:** Filipa Monteiro, Mónica Sebastiana, Maria Salomé Pais, Andreia Figueiredo

**Affiliations:** Plant Systems Biology Lab, Center of Biodiversity, Functional & Integrative Genomics (BioFIG), Science Faculty of Lisbon University, Lisboa, Portugal; Virginia Tech, United States of America

## Abstract

The pivotal role of cultivated grapevine (*Vitis vinifera* L.) in many countries economy is compromised by its high susceptibility to *Plasmopara viticola*, the causal agent of downy mildew disease. Recent research has identified a set of genes related to resistance which may be used to track downy mildew infection. Quantification of the expression of these resistance genes requires normalizing qPCR data using reference genes with stable expression in the system studied. In this study, a set of eleven genes (*VATP16*, *60 S*, *UQCC*, *SMD3*, *EF1α*, *UBQ*, *SAND*, *GAPDH*, *ACT*, *PsaB*, *PTB2*) was evaluated to identify reference genes during the first hours of interaction (6, 12, 18 and 24 hpi) between two *V. vinifera* genotypes and *P. viticola*. Two analyses were used for the selection of reference genes: direct comparison of susceptible, Trincadeira, and resistant, Regent, *V. vinifera* cultivars at 0 h, 6, 12, 18 and 24 hours post inoculation with *P. viticola* (genotype effect); and comparison of each genotype with mock inoculated samples during inoculation time-course (biotic stress effect). Three statistical methods were used, GeNorm, NormFinder, and BestKeeper, allowing to identify *UBQ*, *EF1α* and *GAPDH* as the most stable genes for the genotype effect. For the biotic stress effect, *EF1α*, *SAND* and *SMD3* were the most constant for the susceptible cultivar Trincadeira and *EF1α*, *GAPDH*, *UBQ* for the resistant cultivar Regent. In addition, the expression of three defense-related transcripts, encoding for subtilisin-like protein, *CYP* and *PR10*, was analysed, for both datasets, during inoculation time-course. Taken together, our results provide guidelines for reference gene(s) selection towards a more accurate and widespread use of qPCR to study the first hours of interaction between different grapevine cultivars and *P. viticola*.

## Introduction

Traditional premium cultivars of wine and table grapes are highly susceptible to various diseases, particularly to downy mildew. Grapevine downy mildew is caused by the biotrophic oomycete *Plasmopara viticola* (Berk. et Curt.) Berl. et de Toni. In Europe, disease management became one of the main tasks for viticulture, being the current strategy for downy mildew disease control the massive use of pesticides in each growing season. The introgression of specific genetic traits, such as resistance to pathogens in traditional crops by breeding programs is one of the most promising methods to reduce such disease control measures. The study of this pathosystem has been of great interest and knowledge on resistance genes linked to downy mildew have been inferred from several approaches, such as from transcriptional analysis [1]–[7] to quantitative trait loci (QTL) and linkage map analysis [8]–[14], to loci linked to resistance [3], .

Quantitative real time polymerase chain reaction (qPCR) is currently the most sensitive technique for gene expression analysis due to its reproducibility and sensitivity [18]–[20]. However, qPCR is influenced by a number of variables that strongly interfere with its accuracy and reliability [20]–[22]. qPCR studies require one or more reference genes (RG) as internal controls for the standardization of raw expression data, allowing the correction for variable starting amounts of RNA and for differences in reverse transcription (RT) efficiency, since reference genes are exposed to the same preparation steps as the genes of interest (GOI) [19], [21]–[23]. Reference genes must be validated for each experimental condition [24] and the geometrical averaging of multiple internal control genes should be used [25]. For grapevine, validation of reference genes has been reported for berry development [26], abiotic stress [27] and biotic stress [28],[29]. For grapevine-downy mildew pathosystem, reference genes have been validated for susceptible *V. vinifera* cultivars from 1 to 7 days post- inoculation with *P. viticola*, being V-type proton ATPase (*VATP16*), *60 S* ribosomal protein L18 (*60 S*), ubiquitin conjugating enzyme (*UBQ*) and *SAND* family protein (*SAND*) reported as the most stable [28], [29]. For the first hours of interaction between grapevine and *P. viticola* no reference genes have yet been validated. In this study, we have tested 11 candidate genes for qPCR normalization of gene expression during the first hours of interaction (0 h, 6, 12, 18 and 24 hpi) with *P. viticola*. Two grapevine cultivars with different degrees of resistance towards *P. viticola* were used. Data was analysed to study genotype and biotic stress effects. The best combination of reference genes for each data set was used to assess the expression of three GOIs, pathogenesis-related protein 10, subtilisin-like protease and cyclophilin, known to be induced during downy mildew inoculation [2].

## Materials and Methods

### Plant Material, Experimental Design and *Plasmopora viticola* Inoculation

The grapevine cultivar Regent was selected at the JKI-Institute for Grapevine Breeding Geilweilerhof. It was bred by multiple introgressions from resistant wild genotypes [30], presenting a high degree of resistance to both downy and powdery mildew [31]. Trincadeira is a Portuguese traditional grapevine cultivar widely used for quality wine production and highly sensitive to *Plasmopara viticola* [1]. Both cultivars were propagated under identical greenhouse conditions according to Figueiredo et al. [2]. Briefly, wood cuttings from both grapevine genotypes were harvested at Quinta da Plansel (Montemor-o-Novo, Portugal) and sent to the JKI Institute for Grapevine Breeding (Geilweilerhof, Germany). Wood cuttings were grown in 12 cm diameter pots in Fruhstorfer Erde (soil) Type P at natural day/night rhythm in a temperature range between 5°C and 28°C for 10 weeks. For plant inoculation, *P. viticola* sporangia were collected after an overnight incubation of symptomatic leaves from greenhouse infected plants in a moist chamber at room temperature. Sporangia were carefully recovered by brushing, dried, stored at −25°C and checked for their vitality by microscopy as described in Kortekamp et al. [3]. A suspension containing 10^4^ sporangia ml^−1^ was used to spray the abaxial leaf surface in order to challenge the plants. Mock inoculations with water were also made. After inoculation, plants were kept in a moist chamber (100% humidity) required for optimal infection for 8 h and then under greenhouse conditions at 25°C during the inoculation time course. The third to fifth fully expanded leaves beneath the shoot apex were harvested at 0 h, 6, 12, 18 and 24 hpi, immediately frozen in liquid nitrogen and stored at −80°C. For each genotype, each biological replicate comprehends a pool of three leaves from three different plants. Three independent biological replicates were collected for each cultivar and condition (inoculated and mock inoculated).

### RNA Extraction and cDNA Synthesis

Total RNA was isolated from leaves with the Spectrum™ Plant Total RNA Kit (Sigma-Aldrich, USA) according to the manufacturer’s instructions. Residual genomic DNA was digested with DNase I (On-Column DNase I Digestion Set, Sigma-Aldrich, USA). RNA purity and concentration were measured at 260/280 nm using a spectrophotometer (NanoDrop-1000, Thermo Scientific) while RNA integrity was verified by agarose gel electrophoresis. Genomic DNA (gDNA) contamination was checked by qPCR analysis of a target on the crude RNA [32]. Complementary DNA (cDNA) was synthesized from 2.5 µg of total RNA using RevertAid®H Minus Reverse Transcriptase (Fermentas, Ontario, Canada) anchored with Oligo(dT)_18_ primer (Fermentas, Ontario, Canada), according to manufacturer’s instructions.

### Candidate Gene: Selection and Primer Design

Eleven candidate genes were selected based on previous studies in *Arabidopsis* [33] and grapevine [2], [26], [28], [29], [33], [34]. Nine of these genes were formerly described as reference genes for grapevine downy mildew pathosystem in later inoculation time-points (1–7 days post-inoculation): V-type proton ATPase 16 kDa proteolipid subunit (*VATP16*), 60 S ribosomal protein L18 (*60* *S*), Ubiquinol-cytochrome c reductase complex chaperone (*UQCC*), Small nuclear ribonucleoprotein SMD3 (*SMD3*) from Gamm et al. [28]; Elongation factor 1α (*EF1α*) from Trouvelot et al. [34], Ubiquitin-conjugating enzyme (*UBQ*) and SAND family protein (*SAND*) from Reid et al. [26], glyceraldehyde-3-phosphate dehydrogenase (*GAPDH*) from Selim et al. [29] and Actin (*ACT*) from Figueiredo et al. [2]. The other two gene homologous to *Arabidopis* polypyrimidine tract-binding protein 1 (AT3g01150) and D1 subunit of photosystem I and II reaction centers (ATCg00340) [33], were retrieved from the grapevine TIGR database v. 8 as PTB2 protein (TC109121) and *PsaB* (TC134081), respectively. Grapevine specific primers were designed with Primer Express software version 3.0 (Applied Biosystems, Sourceforge, USA) using the following parameters: amplicon length between 75 and 250 bp; size: 20±2 bp; melting temperature (Tm): 60±2 °C; GC content: ±50%.

### Quantitative Real time PCR

Quantitative RT-PCR (qPCR) experiments were carried out using Maxima™ SYBR Green qPCR Master Mix (2×) kit (Fermentas, Ontario, Canada) in a StepOne™ Real-Time PCR system (Applied Biosystems, Sourceforge, USA). A final concentration of 2.5 mM MgCl_2_ and 0.2 µM of each primer were used in 25 µL volume reactions, together with cDNA as template. The amplification efficiency of each candidate/target gene was determined using a pool representing all cDNA samples. The pool was used to generate a five-point standard curve based on a ten-fold dilution series. Each standard curve was amplified in two independent qPCR runs and each dilution was run in triplicate. Amplification efficiency (E) was calculated from the slope of the standard curve (E = 10^(−1/a)^) where *a* is the slope of the linear regression model (y = a log(x)+ b) fitted over log-transformed data of the input cDNA concentration (y) plotted against quantification cycle (Cq) values (x).

To investigate candidate reference gene stability, cDNA samples were 10-fold diluted. Thermal cycling for all genes started with a denaturation step at 95°C for 10 min followed by 45 cycles of denaturation at 95°C for 15 s and annealing temperatures (Table 1) for 30 s. Each set of reactions included no template control. Dissociation curves and agarose gel electrophoresis were used to analyze non-specific PCR products. Three biological replicates were used for each sample and the experiments were done twice (two technical replicates).

**Table 1:** Candidate reference genes and target genes primer sequences, amplicon length and qPCR analysis.

Gene (Accession Number)*	Predicted function	Primer sequence	Ampliconlength (bp)	Ta (°C)	Tm (°C)	PCR efficiency(E/%)	Regression Coefficient (R^2^)	Average Cq(± SD)
Reference genes								
VATP16 (XM_002269086.1)	Transport	F: CTTCTCCTGTATGGGAGCTG	112	60	82.62	Discarded (unspecific amplification)		
		R: CCATAACAACTGGTACAATCGAC						
UQCC (XM_002264785.1)	unknown	F: CAAAGTATGAGGGTATCCGA	250	60	77.76			
		R: GTATTGCCCAAATTCAACACC						
PTB2	Similar to AT3g01150, involved in RNA binding, nucleic acid and nucleotide binding	F: CGATCCATAACTCGTGCCAAA	113	60	78.79	Discarded (low abundance transcript)		
(TC109121)		R: TGAACCCACCATGAACAACAA						
60 S (XM_002270599.1)	structural constituent of ribosome/translation	F: ATCTACCTCAAGCTCCTAGTC	165	60	79.41	1.932/93.18	0.998	20.20±1.62
		R: CAATCTTGTCCTCCTTTCCT						
SMD3 (AM435088.2)	Pre-mRNA splicing	F: GCTCTGTTGTTGAAGATGGG	156	60	80.35	1.969/96.94	0.994	22.35±1.13
		R: GGAAGCAGTTTGTAGCATCAG						
EF1α (EC959059)	Translation	F: GAACTGGGTGCTTGATAGGC	164	60	79.8	1.992/99.20	0.994	17.37±1.18
		R: ACCAAAATATCCGGAGTAAAAGA						
UBQ (EC922622)	protein degradation.	F: GAGGGTCGTCAGGATTTGGA	75	60	78.78	1.965/96.48	0.994	18.95±1.19
	post-translational modification	R: GCCCTGCACTTACCATCTTTAAG						
SAND (CF405409)	unknown	F: CAACATCCTTTACCCATTGACAGA	76	60	78.78	1.932/93.18	0.996	23.8±1.33
		R: GCATTTGATCCACTTGCAGATAAG						
GAPDH (EF192466)	Glycolysis. gluconeogenesis	F: TCAAGGTCAAGGACTCTAACACC	226	60	81.13	1.936/93.47	0.993	17.12±1.51
		R: CCAACAACGAACATAGGAGCA						
Actin (TC81781**)	Cytoskeletal structural protein	F: ATTCCTCACCATCATCAGCA	89	55	77.44	1.950/94.97	0.996	21.34±1.30
		R: GACCCCCTCCTACTAAAACT						
PsaB	Similar to ATCg00340, involved in chlorophyll binding	F: GGACCCCACTACTCGTCGTATT	148	60	77.06	1.907/90.69	0.998	16.14±2.71
(TC134081)		R: TCCGGAAGTCCACAGAAAAAT						
**Target genes**								
PR10 (HS075818)	Defense	F: GTTTTGACTGACGGCGTTGA	99	62	79.72	1.903/90.31	0.998	15.07±1.38
		R:TGGTGTGGTACTTGCTGGTGTT						
Subtilisin (HS977208)	Defense	F: GTGCTCCAGAGGGACTCGATAT	100	58	76.86	1.967/96.67	0.997	18.63±2.71
		R: TACCTTTCCTTCCACCTTCAACA						
Cyclophilin (CF609761)	Defense/signalling	F: GCCTCTGCACTACAAGGGATCT	96	56	82.34	1.997/99.74	0.991	15.86±1.76
		R: TTCGCCACCAGTACCGTTTC						

SD, standard deviation. * NCBI accession number or TC TIGR number. ** According to Polesani et al. (2010).

### Determination of Reference Gene Expression Stability using GeNorm, NormFinder and BestKeeper

Data analysis was performed in two groups: biotic stress effect and genotype effect. With the biotic stress dataset, gene stability was evaluated by comparing each genotype with mock inoculated control samples at 6, 12, 18 and 24 hpi. With the genotype dataset, gene stability was evaluated when comparing directly the resistant cultivar Regent and the susceptible Trincadeira at 0 h, 6, 12, 18 and 24 hpi.

The stability of candidate reference genes for the different comparison groups was evaluated with BestKeeper [35], GeNorm v. 3.5 [25] and NormFinder [36] tools.

The GeNorm is based on the pairwise variation of a single reference candidate gene relative to all other genes. The main assumption of this approach relies on the expression ratio of the two ideal reference genes being identical in all samples regardless of the conditions tested. GeNorm calculates a gene expression stability measure (M) based on the average pairwise (V) expression ratio between a gene and each of the other genes being compared. Moreover, it performs a stepwise exclusion of the least stable gene and recalculates M until only the two most stably expressed genes are left. A pairwise variation value (V) with a cut-off of 0.15 as threshold is used to select the optimal number of RGs. After, GeNorm estimates the normalization factor (NF*_n_*) using the geometric mean of expression levels of *n* best reference genes [25]. NormFinder is based on a variance estimation approach, which calculates an expression stability value (SV) for each gene analysed. It enables estimation of the overall variation of the reference genes, taking into account intra and intergroup variations of the sample set. According to this algorithm, genes with lowest SV will be top ranked. BestKeeper tool calculates standard deviation (SD) and the coefficient of variation (CV) based on Cq values of all candidate reference genes [35]. The program compares each reference gene to the BestKeeper Index (BKI) and calculates a Pearson’s correlation coefficient (*r*) and *p*-value. Higher correlation coefficients suggest more stable expression. Genes with SD less than 1 and with the highest coefficient of correlation *r* have the highest stability.

A comprehensive ranking considering the 3 algorithms was established by calculating the arithmetic mean ranking value of each RG genes [37]. Each gene was ranked from 1 (most stable) to 8 (least stable) (Table S1).

### Data Analysis and Normalization of *PR10*, *Subtilisin* and *CYP*

Reference gene selection was performed with the three statistical algorithms (GeNorm, NormFinder and Bestkeeper). Cq values from each candidate RG were log-transformed for GeNorm v. 3.5 and NormFinder analysis, while raw Cq values were used for Bestkeeper. After selecting RGs, two normalization strategies were tested: (1) using the 3 top genes given by a comprehensive ranking based on the three methods (GeNorm, NormFinder and BestKeeper); and (2) using the optimal number of RGs based on GeNorm pairwise variation value (the 0.15 cut-off value was followed).

For normalization, Cq values were converted into relative quantities (RQ) by the delta-Ct method [38], incorporating the calculated amplification efficiency (E) for each primer pair [39]. The formula RQ = E^ΔCq^, being the ΔCt calculated as Ct from control samples minus Ct of treated samples was used for both RG and GOI calculations. A normalization factor calculated as the geometric mean of the relative expression of the RGs selected for each normalization strategy was used to obtain the normalized relative quantities (NRQ) [40]. Finally, to obtain GOIs fold expression, the ratio between the RQ values of each gene of interest with NRQ for each normalization strategy was performed.

The expression of three defense-related genes encoding for a pathogenesis related protein 10 (*PR10*, HS075818), a subtilisin-like protease (*subtilisin*, HS977208) and cyclophilin (*CYP*, CF609761) (Table 1) was further analysed in both datasets (genotype and biotic stress). Also, data described by Figueiredo et al. [2] on *PR10*, *CYP* and *Subtilisin* expression at 6 and 12 hpi, allowed to validate the selected RGs for the genotype effect dataset.

Statistical significance (p<0.05) between the two normalization strategies was determined by the Mann-Whitney *U* test using IBM® SPSS® Statistics version 20.0 (SPSS Inc., USA) software.

## Results

### Selection of Candidate Reference Genes and Amplification Specificity

Nowadays, data normalization using a set of reference genes is considered to be the gold standard method for accurate measurement of qPCR expression levels of target transcripts [41]. RNA quality is one of the crucial parameters that must be addressed in a gene expression profiling experiment [42]. In the present study, all samples were analysed spectrophotometrically and in agarose gels showing absorbance ratios at 260/280 and 260/230 nm above 1.8, well-defined bands corresponding to the rRNA and absence of nucleic acid degradation. To confirm the absence of contaminating gDNA, positive and no RT controls were used for each candidate gene amplification. DNase treatment (On-Column DNase I Digestion, Sigma-Aldrich) was followed by a careful check for the absence of gDNA through qPCR analysis of a target on the crude RNA [32].

**Figure 1:**
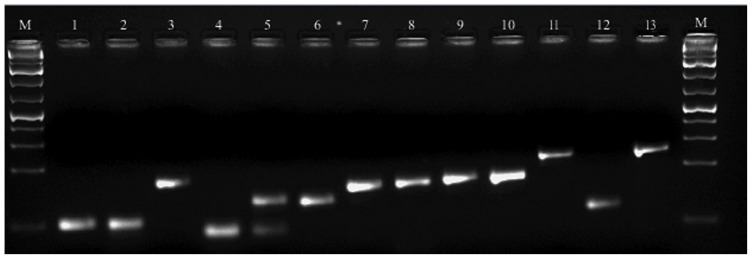
Agarose gel (3%) electrophoresis showing amplicon size for eleven candidate reference genes. M- O’GeneRuler™ 1 kb DNA Ladder Plus (Fermentas), 1- *UBQ*, 2- *SAND*, 3- *ACT*, 4- NTC *VATP16*, 5- *VATP16*, 6- *PTB2*, 7- *PSAB*, 8- *SMD3*, 9- *EF1α*, 10–*60* *S*, 11- *GAPDH*, 12- NTC *UQCC*, 13- *UQCC*.

In qPCR, when using a SYBR Green approach, amplification specificity of several genes should be supported by both melting curve and gel electrophoresis [20]. In our samples, single PCR amplification products with the expected size for each gene were found (Fig. 1). Melting curves of the genes tested were analysed to detect the absence/presence of primer dimer or non-specific PCR products (Fig. S1). For *VATP16* and *UQCC*, melting curves profiles revealed non-specific amplification and primer dimer formation on the amplicon region (Fig. S1). Primers targeting *PTB2PTB2* revealed high Cq values characteristic of low abundance transcript, particularly on inoculated samples of both grapevine genotypes. Thus, *VATP16*, *UQCC* and *PTB2PTB2* were excluded from analysis (Table 1). For all remaining genes, no-template controls (NTCs) had no Cq values or the Cq values ranged between 29 and 34 Cq. Since no amplicon peak was obtained from melting curve analysis, the Cq values observed on NTCs were attributed to primer dimer formation/hairpins, and thus disregarded (Fig. S1).

PCR efficiency of each primer pair was calculated through the standard curve method using the pool of all cDNA samples in a ten-fold serial dilution. The amplification efficiency (E) of the reactions ranged from 1.907 (90.69%) to 1.992 (99.20%), with correlation coefficients R^2^ varying from 0.993 to 0.998 (Table 1). To account that any variation between biological replicates was not due to the treatments but intrinsic to the gene itself, data from the biological replicates were analysed separately by statistical algorithms [43], [44]. The expression stability of the remaining eight candidate genes was evaluated by three different statistical applets: GeNorm, NormFinder and BestKeeper. The analysis was performed for two comparison groups considering genotype and biotic stress effects.

### Biotic Stress Effect

To study the biotic stress effect: each cultivar inoculated with *P. viticola* was compared to a control sample (mock inoculated) at each inoculation time-point (6, 12, 18 and 24 hpi). For *V. vinifera* cv. Trincadeira inoculated with downy mildew, GeNorm ranked *SAND* and *EF1α* (M = 0.881, Table 2) as the most stable genes. NormFinder selected *SMD3* (SV = 0.454) as the most stable gene followed by *EF1α* and *SAND* (SV = 0.722 and SV = 0.727, respectively). Likewise, *SMD3* (SD = 0.66, *r* = 0.76, Table 2) was identified as the most suitable gene for qPCR normalization by BestKeeper analysis, while *EF1 α* (SD = 0.86, *r* = 0.87) and *SAND* (SD = 0.92, *r* = 0.74) were ranked in the third and fifth positions, respectively. Overall, *EF1α*, *SAND* and *SMD3* were the most stable set of genes for *V. vinifera* cv Trincadeira.

**Table 2:** Candidate reference genes for Biotic Stress effect in *V. vinifera* cv Trincadeira calculated by the GeNorm, NormFinder and BestKeeper.

Candidate genes	Trincadeira			
	GeNorm	NormFinder	BestKeeper	
	M	SV	SD	*r*
*SAND*	0.881 (1/2)	0.727 (3)	0.92 (5)	0.74*
*EF1α*	0.881(1/2)	0.722 (2)	0.86 (3)	0.87*
*SMD3*	0.975 (3)	0.454 (1)	0.66 (1)	0.76*
*ACT*	1.038 (4)	0.849 (5)	0.92 (4)	0.75*
*UBQ*	1.088 (5)	1.231 (6)	1.00 (6)	0.58*
*GAPDH*	1.158 (6)	0.813 (4)	0.84 (2)	0.81*
*60* *S*	1.305 (7)	1.511 (7)	1.09 (8)	0.25*
*PsaB*	1.470 (8)	1.799 (8)	1.41 (7)	0.74*

SV. stability value; SD, standard deviation of Cq value; *r*. Pearson coefficient of correlation; * *p≤*0.001. *p*-value associated with the Pearson coefficient of correlation. Ranking order is presented in parenthesis.

For *V. vinifera* cv Regent, *UBQ* and *EF1α* (M = 0.920, Table 3) appeared as the most stable genes considering GeNorm analysis. BestKeeper selected, *GAPDH* (SD = 0.66, *r* = 0.82) as the most stable gene, followed by *EF1α* and *UBQ* (SD = 0.87 and SD 0.92, respectively). Interestingly, *GAPDH* was also found to be the most stable gene with NormFinder analysis. *EF1α* appeared in the third position after *SMD3* (Table 3), while *UBQ* was set in the fourth/fifth position together with *ACT* (SV = 0.869). Considering the three algorithms, *EF1α*, *GAPDH* and *UBQ* were selected as the most suitable RGs for *V. vinifera* cv Regent.

**Table 3:** Candidate reference genes for Biotic Stress effect in *V. vinifera* cv Regent calculated by the GeNorm, NormFinder and BestKeeper.

Candidategenes	Regent			
	GeNorm	NormFinder	BestKeeper	
	M	SV	SD	*R*
*EF1α*	0.920 (1)	0.812 (3)	0.87 (2)	0.91*
*UBQ*	0.920 (2)	0.869 (4/5)	0.92 (3)	0.73*
*SMD3*	1.057 (3)	0.731 (2)	0.53(4)	0.50
*GAPDH*	1.153 (4)	0.579 (1)	0.66 (1)	0.82*
*ACT*	1.184 (5)	0.869 (4/5)	1.01 (6)	0.66*
*SAND*	1.260 (6)	1.476 (6)	1.13 (8)	0.76*
*60* *S*	1.413 (7)	1.509 (7)	1.03 (7)	0.23
*PsaB*	1.711 (8)	2.529 (8)	0.87 (5)	0.36

SV. stability value; SD, standard deviation of Cq value; *r*. Pearson coefficient of correlation;

* *p≤*0.001. *p*- value associated with the Pearson coefficient of correlation. Ranking order is presented in parenthesis.

### Genotype Effect

In order to evaluate the genotype effect on RG stability both cultivars were directly compared prior (0 h) and after inoculation with *P. viticola* (6, 12, 18 and 24 hpi). GeNorm selected, *EF1α* and *UBQ* (M = 0.775) as the two most stable genes (Table 4). The BestKeeper analysis ranked *UBQ* as the most stable gene (SD = 0.77), followed by *GAPDH* (SD = 0.59) and *EF1α* (SD = 0.93). *ACT* was excluded due to a low Pearson correlation (*r* = 0.56, p>0.001, Table S3). The candidate genes *SAND*, *60* *S*, and *PsaB* presented SD values higher than 1 (Table 4) and therefore were considered unstable. NormFinder ranked *UBQ* (SV = 0.581) as the most stable gene, while *GAPDH* (SV = 0.668) and *EF1α* (SV = 0.775) were placed on the second and fourth position, respectively. All three statistical algorithms pointed *UBQ* as the best reference gene for normalizing qPCR genotype data (Table 4).

**Table 4:** Candidate reference genes for Genotype effect as calculated by the GeNorm, NormFinder and BestKeeper programs.

Candidate Genes	GeNorm	NormFinder	BestKeeper	
	M	SV	SD	*r*
*UBQ*	0.775 (1/2)	0.581 (1)	0.77 (1)	0.90*
*EF1α*	0.775 (1/2)	0.775 (4)	0.93 (3)	0.88*
*SAND*	0.939 (3)	1.023 (5)	1.19 (8)	0.85*
*SMD3*	1.077 (4)	0.669 (3)	0.66 (4)	0.56
*GAPDH*	1.118 (5)	0.668 (2)	0.59 (2)	0.74*
*ACT*	1.156 (6)	0.869 (5)	0.85 (5)	0.56
*60* *S*	1.292 (7)	1.617 (7)	1.03 (6)	0.90*
*PsaB*	1.525 (8)	2.144 (8)	1.11 (7)	0.57

SV, stability value; SD, standard deviation of Cq value; *r*, Pearson coefficient of correlation;

* *p≤*0.001. *p*,value associated with the Pearson coefficient of correlation. Ranking order is presented in parenthesis.

### Determination of the Optimal Number of Reference Genes for Normalization by GeNorm

It was suggested that normalization using multiple reference genes gives more accurate results [32]. The GeNorm V value determines the optimal number of RGs to be used, following a 0.15 cut-off value below which the inclusion of an additional reference gene is not required [32].

**Figure 2:**
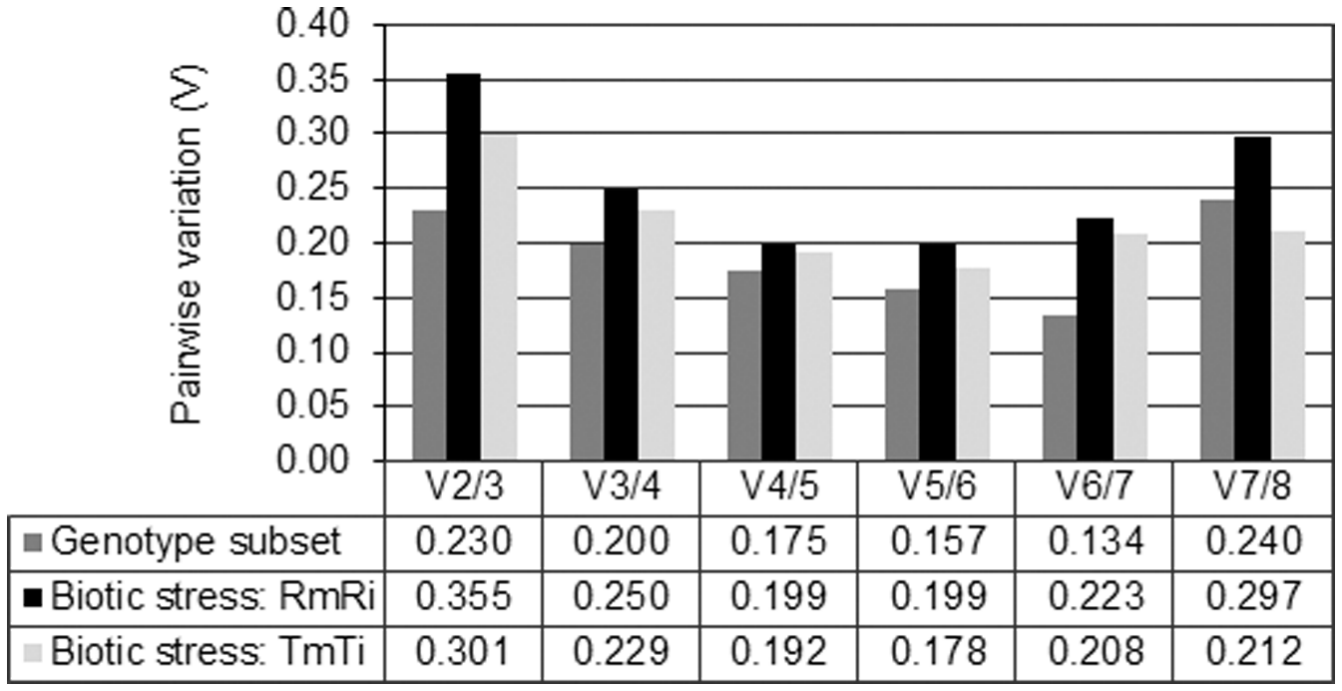
Pairwise variation (V) of candidate genes as predicted by GeNorm. The pairwise variation (Vn/Vn+1) was calculated between the normalization factors NFn and NFn+1. Each pairwise variation value is compared with a recommended cut-off value 0.15, below which the inclusion of an additional reference gene is not required.

The V values were determined for the experimental datasets: biotic stress effect (TmTi: comparison between Trincadeira mock and inoculated samples; RmRi: comparison between Regent mock and inoculated samples) and genotype effect (Fig. 2). The optimal number of reference genes to be used for normalization differed between the analysed datasets. Considering biotic stress, for Trincadeira, the V did not reach the cut-off value (TmTi, Fig. 2), being five genes (V = 0.178) necessary for an accurate normalization, for Regent (RmRi, Fig. 2) four genes are necessary for qPCR normalization (V4/5 and V5/6 = 0.199). Considering the genotype effect, six genes (V6/7 = 0.134) (Fig. 2) should be used to accomplish an accurate qPCR normalization.

### *PR10*, *CYP* and S*ubtilisin* Expression

Two normalization strategies were followed to determine the expression of 3 defense-related genes (*PR10*, *CYP* and *subtilisin*): (1) using the 3 top RGs given by a comprehensive ranking from the three methods (GeNorm, NormFinder and BestKeeper), and (2) using the optimal number of reference genes selected by the GeNorm V value for each condition studied (pairwise variation analysis, Fig. 2).

#### Biotic stress

**Figure 3:**
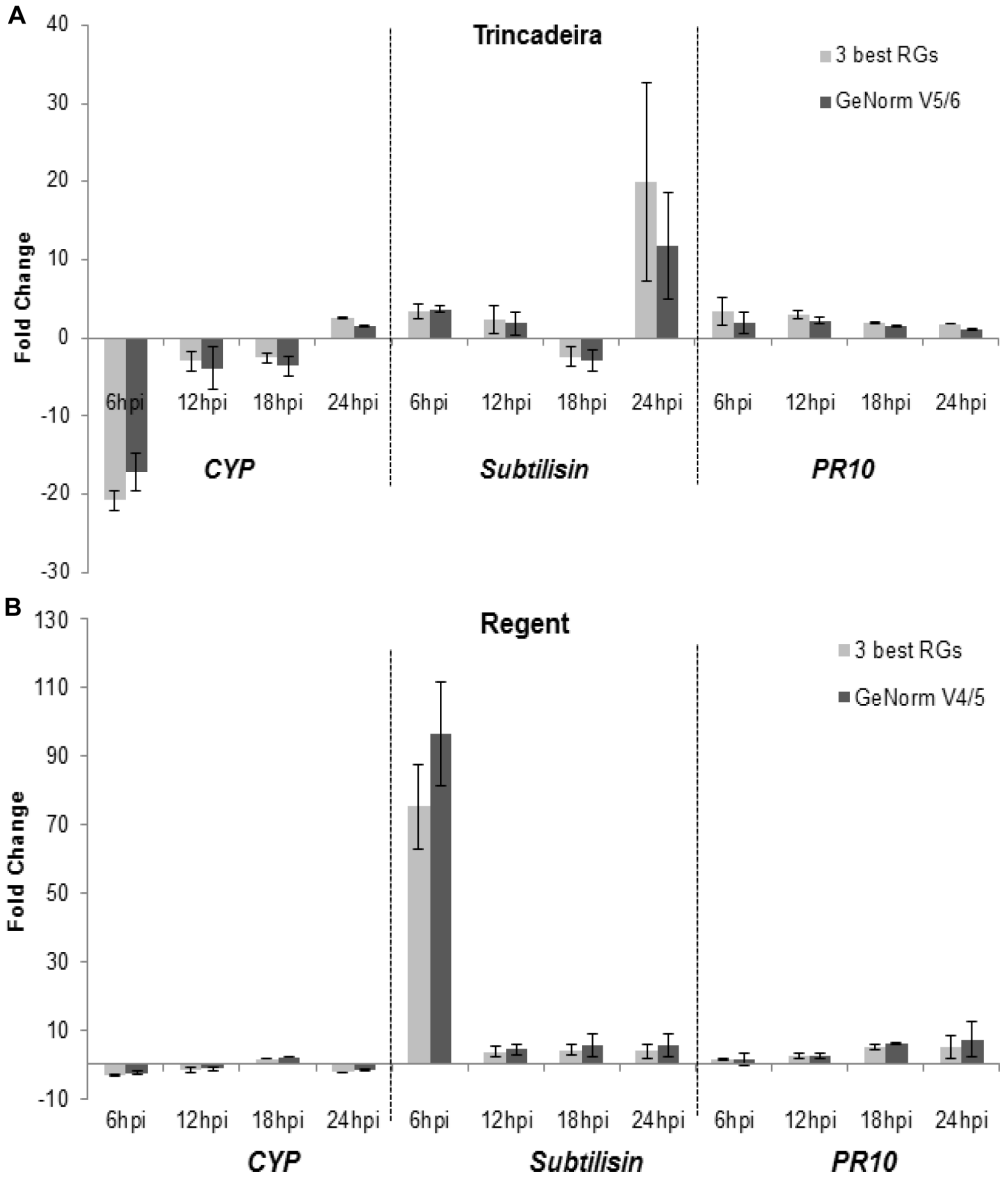
*CYP*, *subtilisin*, *PR10* expression in Biotic Stress effect when comparing independently Trincadeira (a) and Regent (b). Two normalization strategies are presented. Fold Change: expression of inoculated leaves (6, 12, 18 and 24 hpi) divided by mock inoculated samples. Asterisks indicate significant differences between each normalization strategy. RG, reference gene. Median and MAD (mean absolute deviation) values of three biological replicates are presented.

For *V. vinifera* cv Trincadeira, *EF1α*, *SAND* and *SMD3* were selected as the three top ranked genes for normalization by the combination of the three methods. Additionally, the top five ranked *EF1α*, *SAND*, *SMD3*, *ACT* and *UBQ* genes from GeNorm pairwise analysis were also tested for qPCR normalization (Fig. 3A).

In *V. vinifera* cv Regent, *EF1*α, *GAPDH* and *UBQ* were used as the three top ranked genes, while *EF1α*, *UBQ*, *SMD3* and *GAPDH* genes retrieved from the pairwise analysis (V4/5) were tested as a second normalization strategy (Fig. 3B). For both genotypes, the expression profile of the different GOIs tested was similar using the two normalization approaches (Fig. 3A, B). *CYP* appeared downregulated until 24 hpi in Trincadeira, while in Regent it is downregulated until 18 hpi and at 24 hpi the expression decreases again. *Subtilisin* was upregulated in both genotypes, showing the greater fold change in Regent at 6 hpi and in Trincadeira at 24 hpi. *PR10* was equally over-expressed in both genotypes during downy mildew infection time-course.

#### Genotype effect

**Figure 4:**
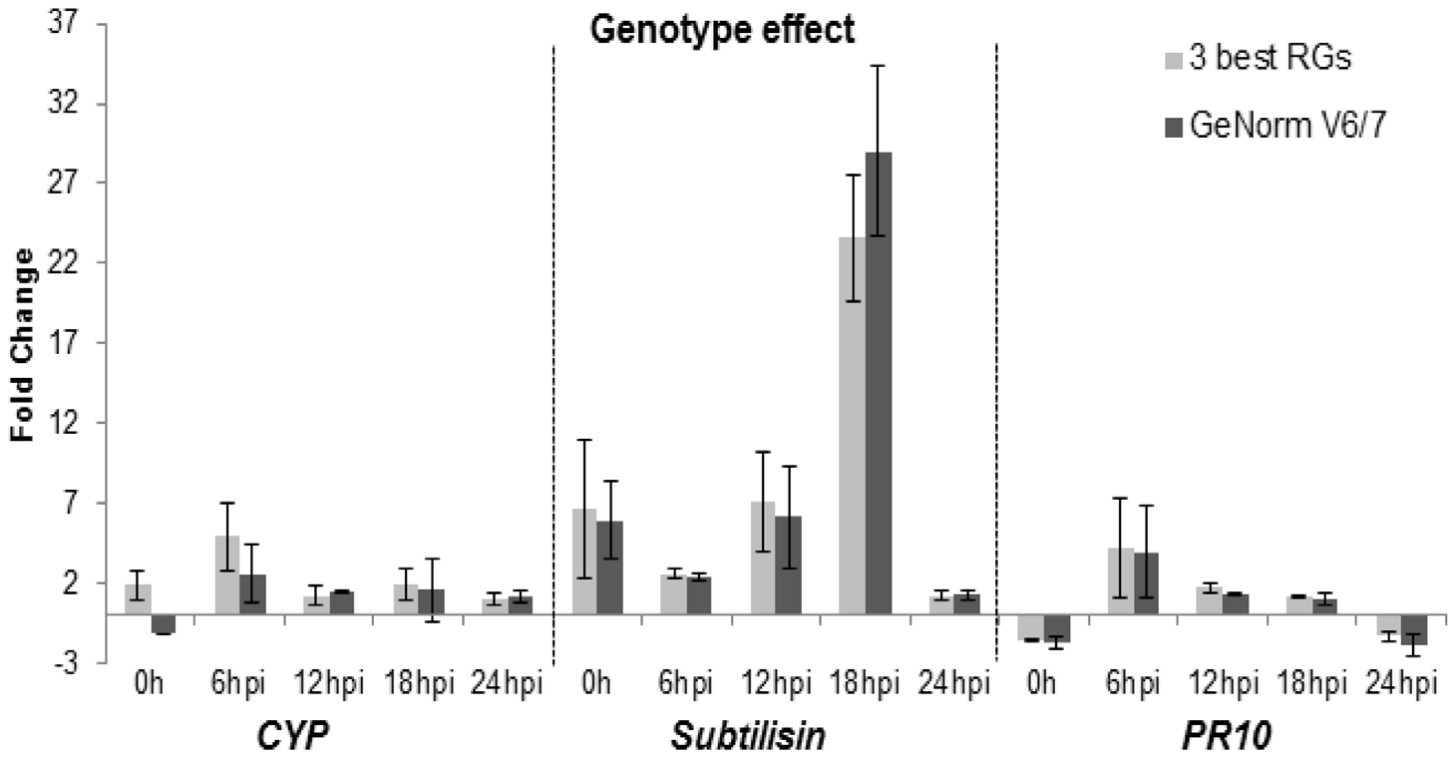
Fold change variation when comparing Regent and Tricadeira prior and during inoculation time-course for *subtilisin*, *CYP* and *PR10*. Two normalization strategies are presented. Fold Change: expression of Regent samples divided by Trincadeira samples. Asterisks indicate significant differences between each normalization strategy. RG, reference gene. Median and MAD (mean absolute deviation) values of three biological replicates are presented.

*UBQ*, *EF1α* and *GAPDH* were selected as RGs from a combined analysis using GeNorm, NormFinder and BestKeeper. The GeNorm pairwise analysis (Fig. 2) selected *UBQ*, *EF1α*, *SAND*, *SMD3*, *GAPDH* and *ACT* for normalization (Table 4). Overall, the *CYP*, *subtilisin* and *PR10* expression profile was similar with the two normalization strategies tested, with the exception of *CYP* at 0 h (Fig. 4). In the resistant cultivar there is an upregulation of the expression of the three GOIs after *P. viticola* inoculation, when compared to Trincadeira (Fig. 4). Interestingly, *subtilisin* is constitutively more expressed in Regent than in Trincadeira (0 h, Fig.4).

## Discussion

Normalization is one of the key factors affecting the accuracy and reliability of quantitative gene expression analysis. Here, we describe an assessment of eleven reference genes for their use as internal controls in gene expression studies for the first hours of interaction between grapevine and *Plasmopara viticola*. Two datasets were used representing two different experimental designs: the first dataset compares each genotype with control samples (mock inoculated) during inoculation time-course (biotic stress effect), while the second dataset compares directly a resistant and a susceptible cultivar prior and after *P. viticola* inoculation evaluating the genotype effect on plant defence response.

Studies using a combination of GeNorm, BestKeeper, and NormFinder for selecting reference genes have described minor to substantial discrepancies in the results of the three programs, which may be easily explained by the different mathematical models associated with each approach [26], [45]. In our study, no substantial differences were obtained in the ranking of candidate reference genes when using the three statistical algorithms. A comprehensive ranking considering the three algorithms was performed and results revealed to be consistent with those of GeNorm analysis, only differing on the ranking orders of the most stable genes (Table S1). Some of the candidates were repeatedly ranked in the last positions, regardless the dataset under analysis: *PsaB* and *60* *S*. *PsaB*, is the *Vitis* homolog of the *Arabidopsis* ATCg00340 *locus*, which was previously reported as a potential RG in biotic stress studies [33], revealed to be unsuitable for our experiment. Also, *60* *S* was not stable for the first hours of interaction between the susceptible *V. vinifera* cv. Trincadeira and *P. viticola*, despite being previously reported as one of the most stable genes for susceptible *V. vinifera* cv. Marselan leaves inoculated with *P. viticola* from 1 to 7 days [28]. The early time-points used in our study may account for this difference. In our study and regardless the genotype, *EF1α* was selected as the most stable gene for the first hours of grapevine-downy mildew interaction. *EF1α* was also selected as the most stable gene for late blight infection of potato [46]. However, recent studies with *V. vinifera* cv Riesling inoculated with downy mildew pointed *EF1α* as one of the least stable reference genes in a 1–5 dpi time-course experiment [29]. These results reinforce the need for systematic selection of stable reference genes for each experimental condition tested.

Several studies have been performed on grapevine resistance response towards *P. viticola*, transcripts as *PR10*, subtilisin-like protein and *CYP* have been pointed out as defense and signalling candidate genes for downy mildew resistance [1], [2], [4], [5], [7]. Subtilisin-like proteins seem to be involved in pathogen recognition leading to further induction of defense responses [47]–[52]. Cyclophilins have been shown to accumulate upon fungal infection [53], [54] and to play an important role in signal transduction under stressful conditions [55]. PR10 family is one of the most important proteins in response to fungal invasion [56] and on other biotic or abiotic stresses [57].

Considering the genotype effect, a recent study by Figueiredo et al. [2] allowed us to validate the RGs selected at 6 and 12 hpi. When comparing Regent to Trincadeira, *PR10*, *subtilisin* and *CYP* are over-expressed in Regent at 6 and 12 hpi which is in accordance with [2]. The expression of these three genes was also accessed for the remaining time-points. During inoculation time-course, for *PR10, CYP* and *subtilisin* expression, both normalization strategies showed the same trend: upregulation in the resistant cultivar (Fig. 4). Prior to inoculation (0 h), *subtilisin* expression is consistent to [1], however different expression was obtained with the two normalization strategies for *CYP* expression, upregulation using the three best genes and downregulation with the pairwise analysis selected genes (Fig. 4). As *CYP* expression at 0 h using the 3 best RGs is consistent with [1], we may consider that, in our study the normalization strategy using the 3 best RGs is more accurate.

Considering the biotic stress effect, *PR10* appeared upregulated in both genotypes, when compared to mock inoculated samples, during inoculation time-course, which is in accordance to previous works [4],[5]. Cyclophilin was downregulated in both genotypes at 6 and 12 hpi. At 18 hpi, a1.71-fold increase of *CYP* in Regent was obtained (Fig. 3B), while in Trincadeira a 2.6-fold increase only occurred at 24 hpi (Fig. 3A). As cyclophilins were shown to play a role in signal transduction under stressful conditions [55], the earlier expression peak (18 hpi) observed in the resistant genotype (Regent) may be associated to a faster pathogen recognition and/or defense response activation. Subtilisin-like protein was highly transcribed in Regent at 6 hpi when compared to the control samples, while in Trincadeira the increased in transcription only occurred at 24 hpi. We may hypothesize, as suggested by Figueiredo et al. [2], that subtilisin is participating in pathogen recognition and on the activation of the defence response. As a result, Regent could recognize the pathogen and thus reacts to its invasion faster than Trincadeira, which may account for its increased resistance towards this pathogen [2].

A nonparametric test was used to compare the two normalization strategies (Table S2) in order to determine significant differences in the expression of the GOIs. As no statistically differences were found, we may assume that for an accurate normalization, in our experimental conditions, the top three RGs selected by the combination of GeNorm, NormFinder and BestKeeper are appropriate. In summary, *UBQ*, *EF1α* and *GAPDH* should be used when directly comparing Regent to Trincadeira prior and during inoculation time-course (genotype effect), *EF1α*, *SAND* and *SMD3* should be used for Trincadeira data normalization and *EF1α*, *GAPDH* and *UBQ* for Regent data normalization (Biotic stress effect). The proposed RGs are a valuable genomic source to study the early kinetics of plant-pathogen interaction and could be a good starting point for gene expression studies for other grapevine genotypes with similar response to downy mildew. Our results clearly showed that RGs are genotype-dependent and that different RGs should be used for normalization of qPCR studies in compatible and incompatible interactions.

## Supporting Information

Figure S1 **Primer specificity test through dissociation curve analysis collected from StepOne™ software ver. 2.2.2 (Applied Biosystems).** *UBQ* (A), *SAND* (B), *ACT* (C), *VATP16* (D), *PTB2* (E), *PsaB* (F), *SMD3* (G), *EF1α* (H), *60 S* (I), *GAPDH* (J) and *UQCC* (K). Non-template control is indicated by a black arrow.
(PDF)

Table S1 **Comprehensive ranking of the candidate RGs calculated as the arithmetic mean ranking value of each gene using the three applets.** Genes were ranked from the most stable (1) to the least stable (8).
(XLS)

Table S2 **Test statistics and Ranks given by the Mann-Whitney** ***U*** **test on** ***PR10*** **,** ***subtilisin*** **and** ***CYP*** **expression, comparing the two normalization strategies followed.**
(XLSX)

Table S3 **Descriptive statistics of reference gene expression in all datasets analysed based on the BestKeeper approach.**
(PDF)
