# Supplementary figures and images for: Reference Gene Selection and Validation for the Early Responses to Downy Mildew Infection in Susceptible and Resistant Vitis vinifera Cultivars
Source: PLoS One. 2013 Sep 4;8(9):e72998. doi: 10.1371/journal.pone.0072998 (PMC3762845; doi:10.1371/journal.pone.0072998)

(A) UBQ

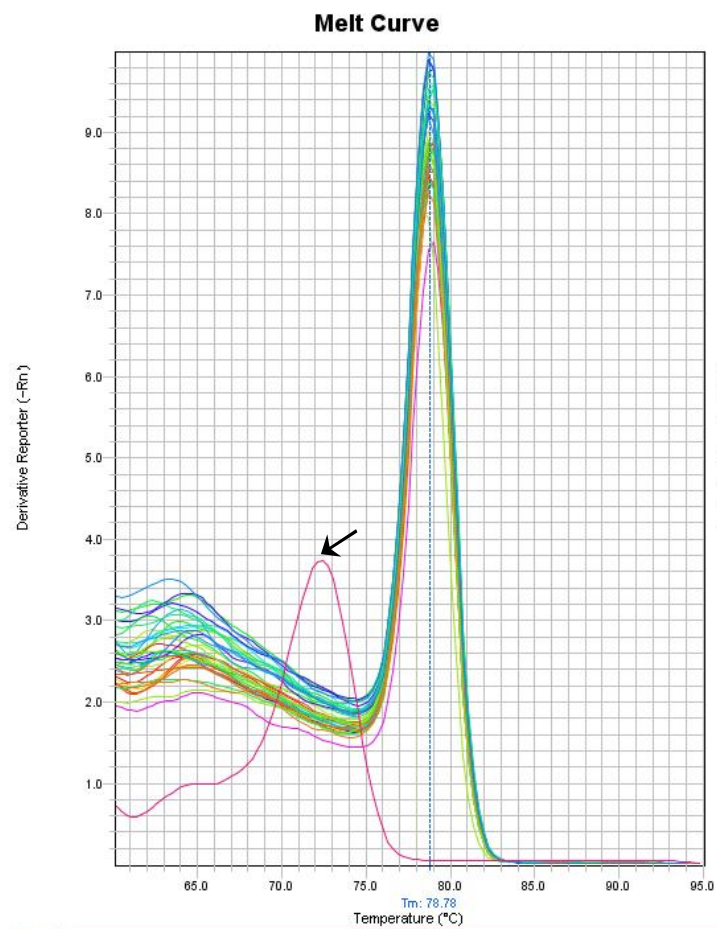

(B) SAND

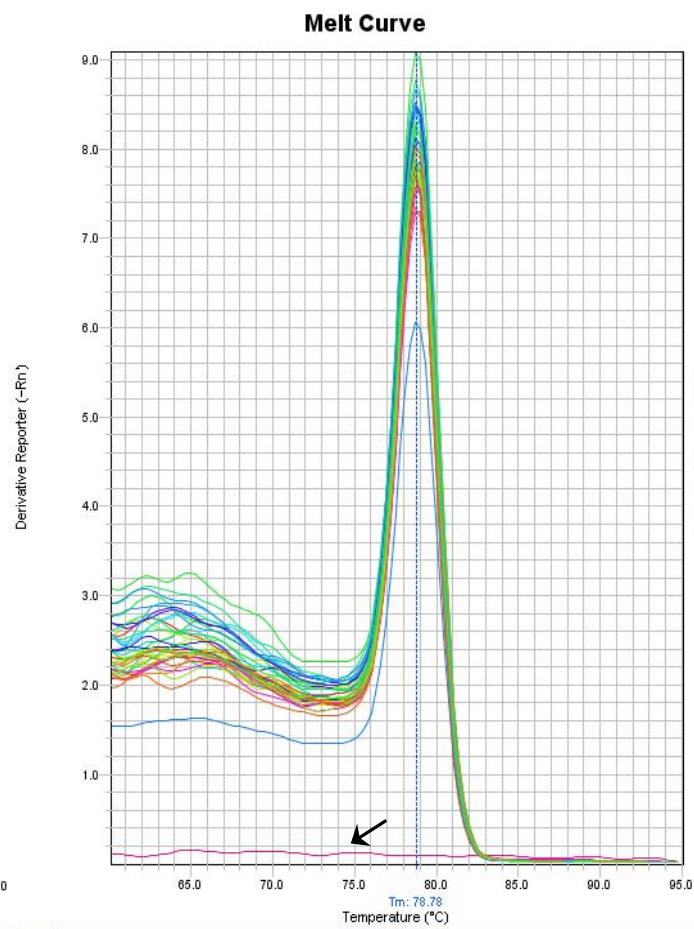

(C) ACT

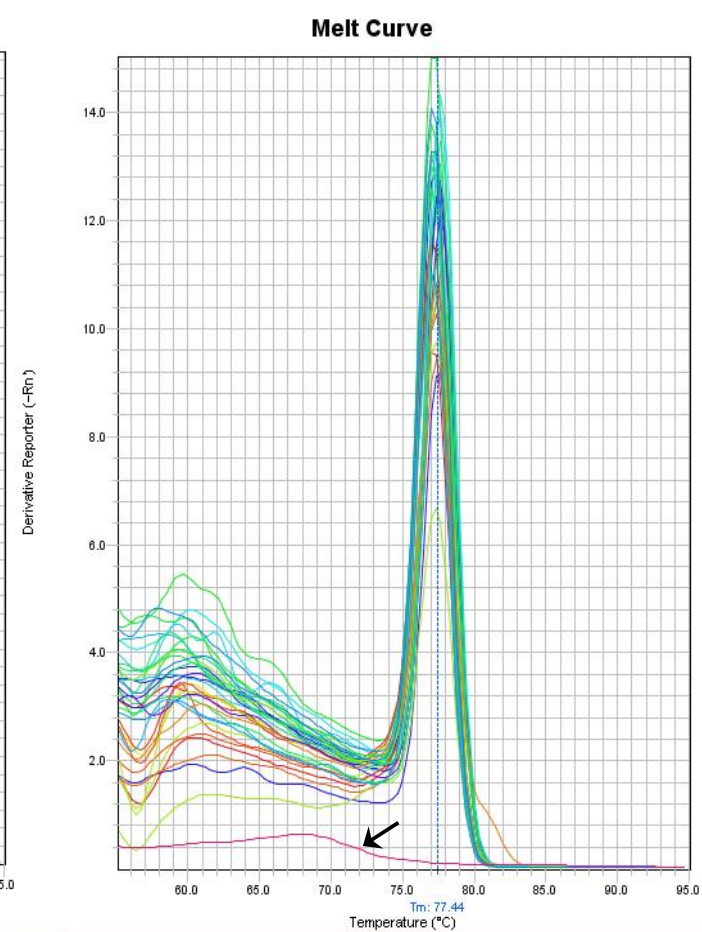

(D) VATP16

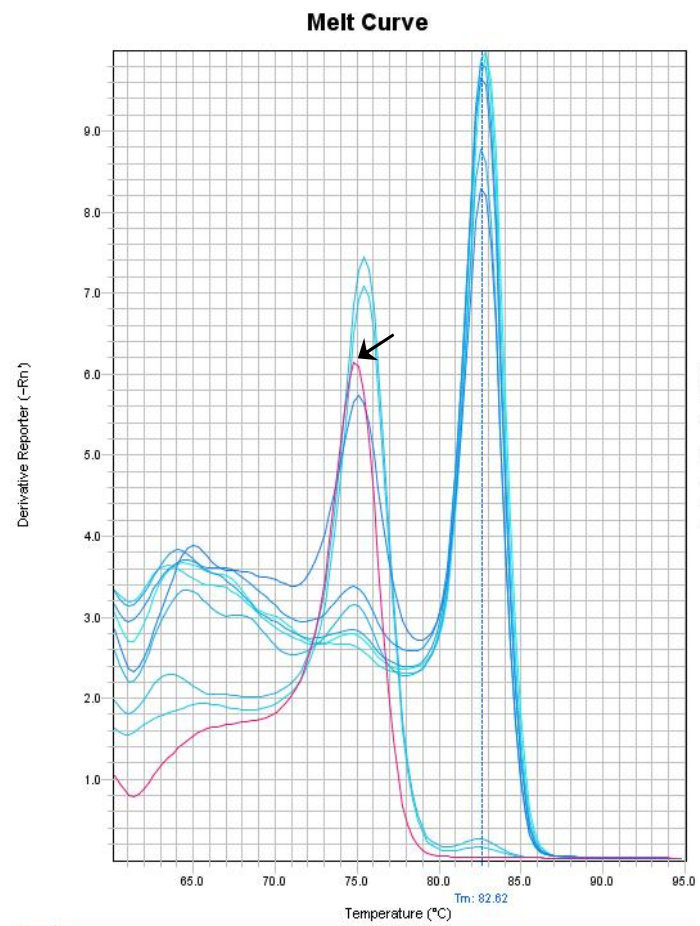

(E) PTB2

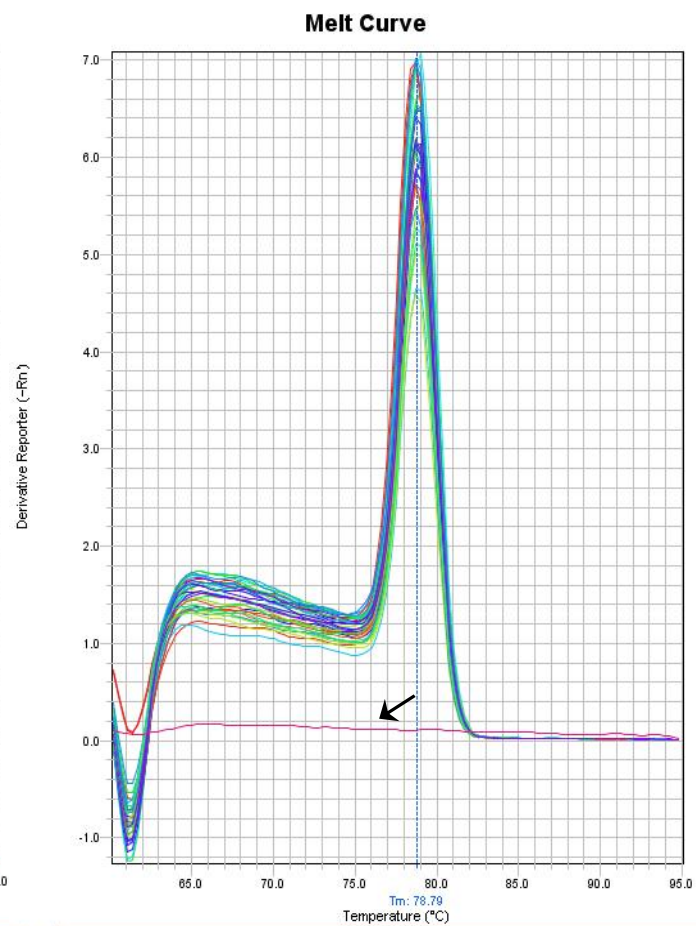

(F) PsaB

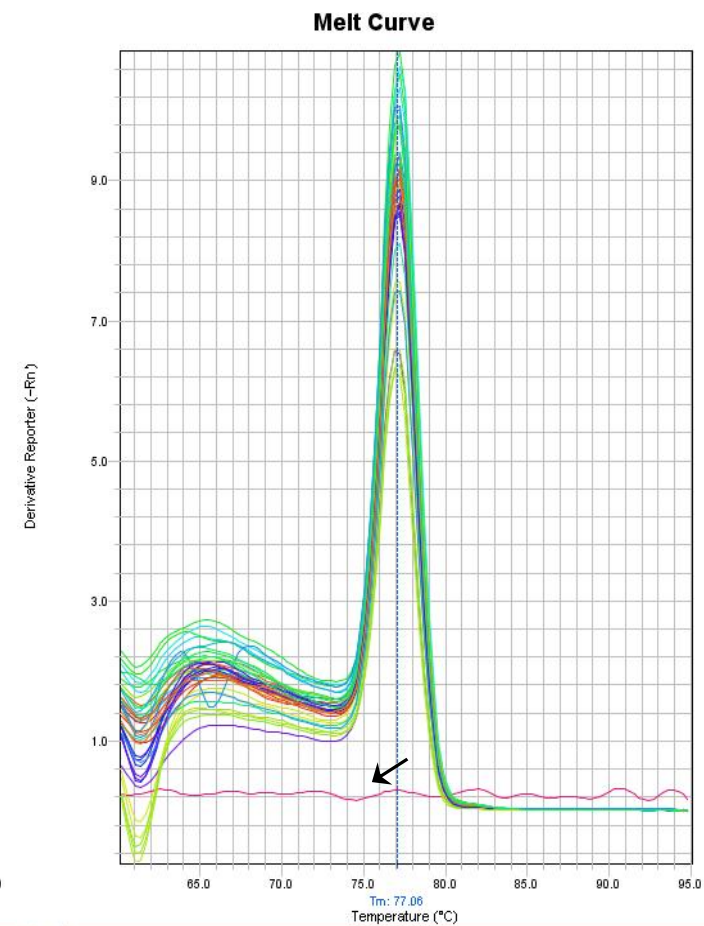

(G) SMD3

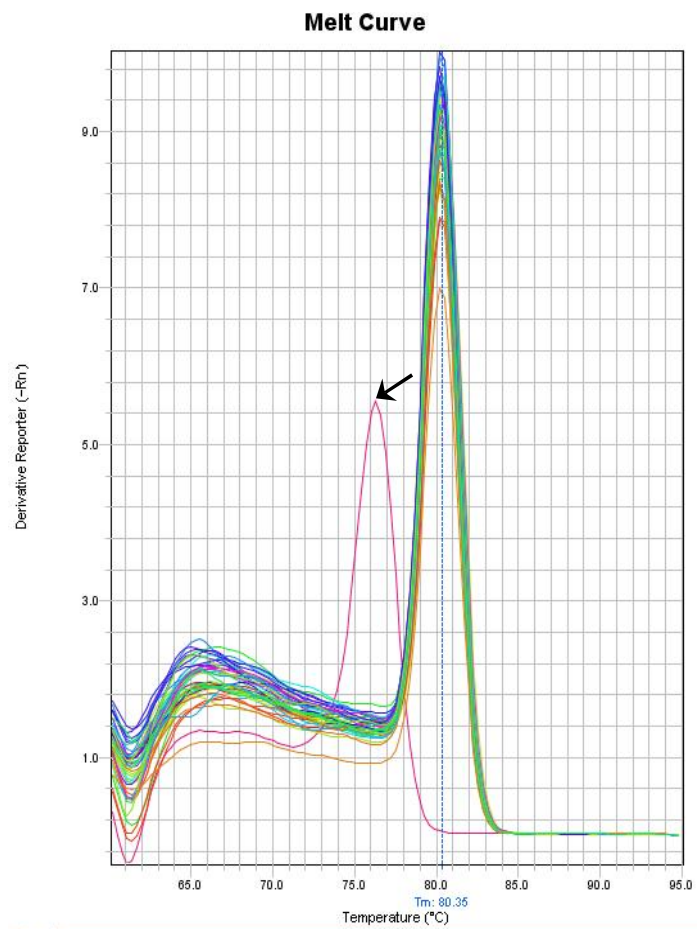

(H) EF1 $\alpha$

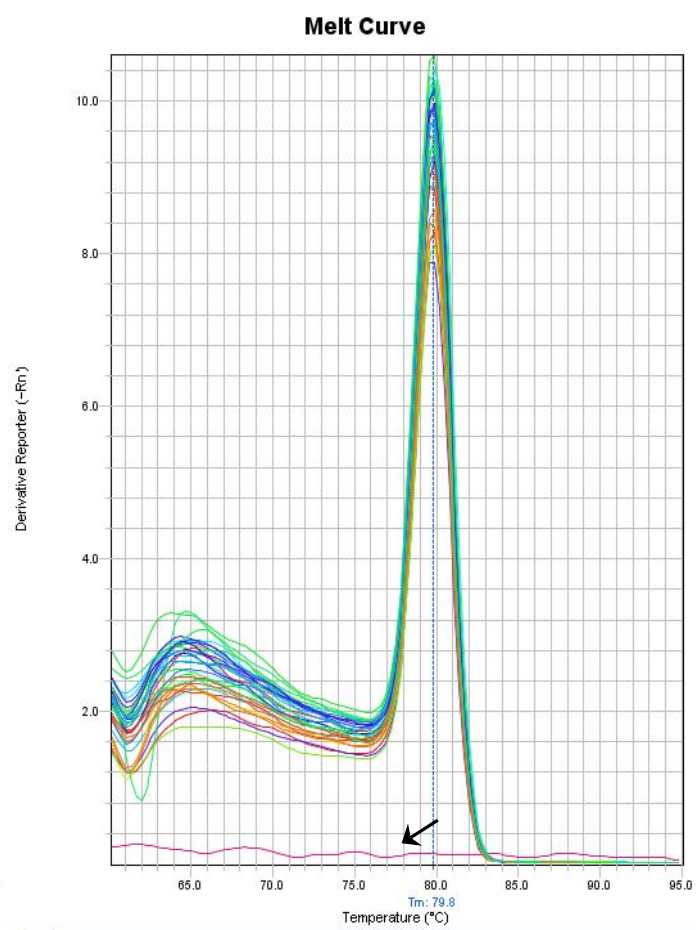

(I) 60S

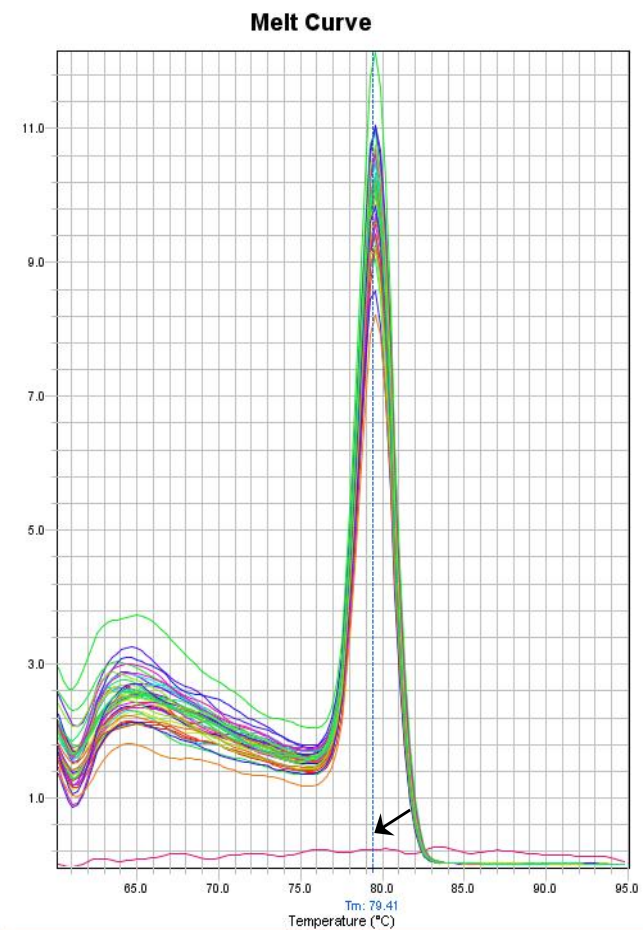

## (J) GAPDH

### Melt Curve

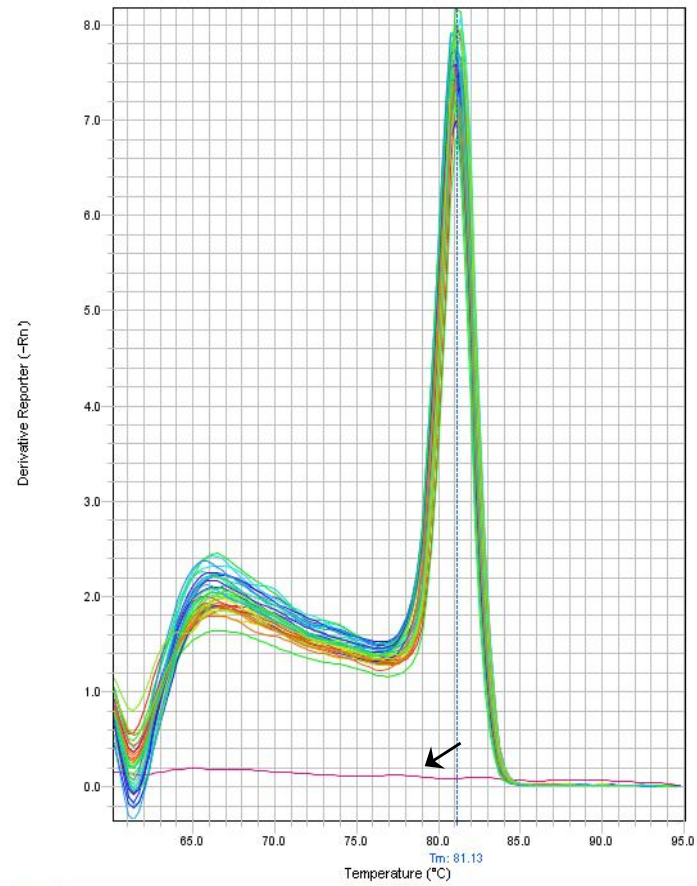

## (K) UQCC

### Melt Curve

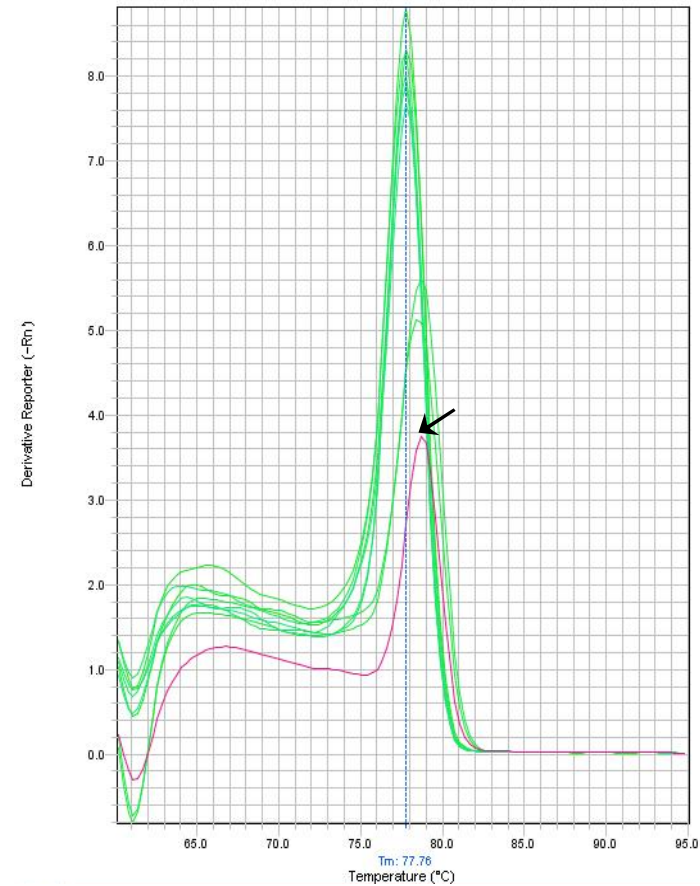

Supplement: Figure S1 — Primer specificity test through dissociation curve analysis collected from StepOne™ software ver. 2.2.2 (Applied Biosystems). UBQ (A), SAND (B), ACT (C), VATP16 (D), PTB2 (E), PsaB (F), SMD3 (G), EF1α (H), 60 S (I), GAPDH (J) and UQCC (K). Non-template control is indicated by a black arrow. (PDF) [file pone.0072998.s001.pdf]
