# Supplementary material for: Reference Gene Selection and Validation for the Early Responses to Downy Mildew Infection in Susceptible and Resistant Vitis vinifera Cultivars
Source: PLoS One. 2013 Sep 4;8(9):e72998. doi: 10.1371/journal.pone.0072998 (PMC3762845; doi:10.1371/journal.pone.0072998)
Supplement: Table S3 — Descriptive statistics of reference gene expression in all datasets analysed based on the BestKeeper approach. (PDF) [file pone.0072998.s004.pdf]

- Descriptive statistics of reference gene data in genotype subsets (0h, 6, 12, 18 and 24 hpi Trincadeira and Regent) based on the BestKeeper approach

	**UBQ**	**SMD3**	**SAND**	**PsaB**	**GAPDH**	**EF1α**	**ACT**	**60S**
n	25	25	25	25	25	25	25	25
GM [CP]	18.21	22.17	23.32	15.46	16.45	17.02	20.57	20.14
AM [CP]	18.23	22.19	23.37	15.54	16.47	17.06	20.59	20.18
min [CP]	16.03	20.25	20.29	12.44	15.00	14.38	18.98	18.20
max [CP]	19.82	24.46	26.08	18.36	18.13	18.85	22.97	22.73
SD [± CP]	0.77	0.66	1.19	1.11	0.59	0.93	0.85	1.03
CV [% CP]	4.23	2.98	5.10	7.16	3.60	5.43	4.12	5.11
coeff. of corr. [r]	0.900	0.560	0.854	0.569	0.744	0.884	0.563	0.900
p value	0.001	0.004	0.001	0.003	0.001	0.001	0.003	0.001

n. number of cDNA samples; GM. geometric mean of Cq value; AM. arithmetic mean of Cq value; min and max. extreme values of Cq; SD [± CP]. standard deviation of Cq value; CV [% CP]. coefficient of variance expressed as percentage of Cq value; r. Pearson coefficient of correlation; *p* value. p value associated with the Pearson coefficient of correlation.

- Descriptive statistics of reference gene data in inoculated (6, 12, 18, 24 hpi) and mock inoculated Trincadeira based on the BestKeeper approach

	**UBQ**	**SMD3**	**SAND**	**PsaB**	**GAPDH**	**EF1α**	**ACT**	**60S**
n	23	23	23	23	23	23	23	23
GM [CP]	18.88	22.59	23.84	15.40	16.58	17.33	21.13	19.93
AM [CP]	18.92	22.61	23.86	15.50	16.61	17.37	21.16	19.97
min [CP]	17.21	21.33	21.67	12.67	14.71	15.16	19.37	18.16
max [CP]	21.40	24.46	25.46	19.62	19.48	18.85	23.60	22.85
SD [± CP]	1.00	0.66	0.92	1.41	0.84	0.86	0.92	1.09
CV [% CP]	5.31	2.92	3.85	9.06	5.08	4.98	4.37	5.48
coeff. of corr. [r]	0.575	0.759	0.744	0.741	0.814	0.873	0.746	0.249
p value	0.001	0.001	0.001	0.001	0.001	0.001	0.001	0.251

n. number of cDNA samples; GM. geometric mean of Cq value; AM. arithmetic mean of Cq value; min and max. extreme values of Cq; SD [± CP]. standard deviation of Cq value; CV [% CP]. coefficient of variance expressed as percentage of Cq value; r. Pearson coefficient of correlation; *p* value. p value associated with the Pearson coefficient of correlation.

- Descriptive statistics of reference gene data in inoculated (6, 12, 18, 24 hpi) and mock inoculated Regent based on the BestKeeper approach

	**UBQ**	**SMD3**	**SAND**	**PsaB**	**GAPDH**	**EF1α**	**ACT**	**60S**
n	23	23	23	23	23	23	23	23
GM [CP]	18.62	21.53	23.54	15.14	16.68	17.04	20.85	19.66
AM [CP]	18.65	21.54	23.59	15.18	16.70	17.08	20.89	19.70
min [CP]	16.03	20.25	20.29	12.44	15.00	14.38	18.98	18.18
max [CP]	20.67	22.92	26.08	18.04	18.38	19.47	23.88	22.73
SD [± CP]	0.92	0.53	1.13	0.87	0.66	0.87	1.01	1.03
CV [% CP]	4.92	2.44	4.80	5.70	3.93	5.09	4.83	5.22
coeff. of corr. [r]	0.729	0.497	0.760	0.361	0.816	0.906	0.662	0.232
p value	0.001	0.022	0.001	0.107	0.001	0.001	0.001	0.311

n. number of cDNA samples; GM. geometric mean of Cq value; AM. arithmetic mean of Cq value; min and max. extreme values of Cq; SD [± CP]. standard deviation of Cq value; CV [% CP]. coefficient of variance expressed as percentage of Cq value; r. Pearson coefficient of correlation; *p* value. p value associated with the Pearson coefficient of correlation.
